# Prevention of chemotherapy-induced nausea and vomiting in the real-world setting in Spain

**DOI:** 10.1007/s12094-021-02623-8

**Published:** 2021-05-06

**Authors:** Y. Escobar Álvarez, J. De Castro Carpeño, D. Bell, A. Drago, A. Franceschetti

**Affiliations:** 1 Department of Medical Oncology, Gregorio Marañón University General Hospital, C/ Dr. Esquerdo, 46, 28007 Madrid, Spain; 2 Department of Medical Oncology, La Paz University Hospital, Madrid, Spain; 3 Ipsos Healthcare, London, UK

**Keywords:** Chemotherapy-induced nausea and vomiting, Highly emetogenic chemotherapy, Antiemetics, NK_1_RA-based regimens, MASCC/ESMO guidelines adherence

## Abstract

**Purpose:**

Proper monitoring and management of chemotherapy-induced nausea and vomiting (CINV) with antiemetics is crucial for cancer patients. This study aimed to evaluate the use of antiemetics for the treatment of highly emetogenic chemotherapy (HEC) including carboplatin in the real-world setting in Spain.

**Methods:**

A representative panel of cancer specialists was asked to collect information about the antiemetic treatments provided to patients receiving chemotherapy. Records formed part of the Global Oncology Monitor^©^ database (Ipsos Healthcare, London, UK). Chemotherapy data were extrapolated using Ipsos Healthcare’s projection methodology.

**Results:**

A total of 73 experts were finally included. Data from 9519 patients, estimated to be representative of 202,084 patients, were collected. HEC (and carboplatin-based chemotherapy) was administered to 73,118 (36%) patients, cisplatin-based therapy being the most frequent treatment (*n* = 34,649, 47.38%). Neurokinin-1 receptor antagonists (NK_1_RAs) alone or in combination were used as prophylaxis for CINV in 14,762 (20%) patients, while the combination of NK_1_RA with 5-hydroxytryptamine-3 receptor antagonist (5-HT_3_RAs) and dexamethasone as recommended by the international guidelines was used in 5849 (8%) patients only. No antiemetic prophylaxis was administered to 8.46% of the patients receiving HEC (*n* = 6189). Physicians classified cisplatin-, anthracycline-cyclophosphamide (AC-), and carboplatin-based regimens as HEC in 63%, 22% and 4% of the cases, respectively.

**Conclusions:**

The use of NK_1_RA-containing regimens for CINV prevention in patients treated with HEC was less than expected, suggesting poor adherence to international antiemetic guidelines.

## Introduction

Despite advances in symptom management, chemotherapy-induced nausea and vomiting (CINV) remains one of the most distressing side effects among patients undergoing systemic anticancer therapy [1, 2], negatively impacting not only their quality of life [3] but also their therapeutic compliance.

CINV may be classified into three categories based on when it develops after chemotherapy administration: acute, occurring within the first 24 h; delayed, identified between 24 and 120 h after chemotherapy treatment; and anticipatory, before a treatment as a conditioned response to CINV in previous cycles [4]. Thus, monitoring CINV is crucial from the start of chemotherapy, because early prevention reduces the risk in subsequent chemotherapy cycles [5] and indirectly increases overall survival [6, 7]. Following antiemetic guidelines has also demonstrated a 10% improvement in the degree of CINV control [8].

International antiemetic guidelines agree that prophylaxis of CINV must be the main objective of antiemetic therapy and should be determined on the basis the emetogenicity of the chemotherapy, CINV history, and individual risk factors [4]. Thus, prophylaxis should be implemented in patients with a risk of CINV of 10% or greater and cover the entire risk period [4]. Otherwise, it is worth knowing that patients will have to face this problem during their treatment. Guidelines report similar efficacy for oral and intravenous (IV) administration routes. In patients receiving highly emetogenic chemotherapy (HEC), including cisplatin and the anthracycline–cyclophosphamide [AC] combination [9, 10], with a > 90% risk for emesis, the Multinational Association of Supportive Care in Cancer/European Society for Medical Oncology (MASCC/ESMO) [11, 12], the National Comprehensive Cancer Network (NCCN) [13], and the American Society of Clinical Oncology (ASCO) [14] all recommend prophylaxis with a 5-hydroxytryptamine-3 receptor antagonist (5-HT_3_RA), a neurokinin-1 receptor antagonist (NK_1_RA), and dexamethasone (DEX). Alternatively, a quadruplet regimen involving the addition of olanzapine to NK_1_RA + 5-HT_3_RA + DEX is recommended [13, 15]. NK_1_RAs approved for the prevention of CINV include aprepitant (oral) and fosaprepitant (IV) in combination with 5-HT_3_RA–DEX, or the fixed-combination agent NEPA (oral), comprising the NK1RA netupitant and the 5-HT_3_RA palonosetron, combined with dexamethasone alone [16]. The intravenous NEPA formulation was approved in the European Union in early 2020.

The aim of this study was to evaluate the use of antiemetic treatments in the prophylaxis of HEC (and carboplatin-based chemotherapy) in the real-world setting.

## Methods

### Study design and inclusion criteria

A representative sample of Spanish experts was screened. To be included, cancer specialists had to be primary therapeutic decision makers and treat ≥ 5 cancer patients per month with anticancer drug therapy. A chemotherapy treatment was defined as at least one dose of a cytotoxic anticancer drug.

### Data collection

The representative panel of experts completed forms directly from patients’ medical charts, collecting information such as patient demographics, diagnosis, staging, full previous and current treatment history, and supportive care. They were instructed to provide information on their last patients seen who were currently receiving an anticancer treatment. Each participating physician was asked to provide up to ten records per month to avoid bias in data collection. Physicians with larger practices supplied more records than those with smaller practices. Furthermore, up to four physicians could participate from the same institution if it was a large cancer centre. Otherwise, no more than two physicians per practice were allowed. Finally, panellists were not required to complete the maximum number of records each month or to participate every month.

Records formed part of the Global Oncology Monitor^©^ database (Ipsos Healthcare, London, UK), a retrospective medical chart review that contains real-world prescribing information for all types of tumours retrieved from patients’ clinical records from 20 different countries, with data in some countries extending back over 20 years. For this study, treatment-related data collected in Spain by physicians from patients’ charts between January and December 2018 were compiled.

### Data analyses

Chemotherapy data were extrapolated based on the total number of physicians who treat their patients with chemotherapy. Sample bases were projected up using Ipsos Healthcare’s projection methodology in which each patient has its own unique weight based on individual characteristics (i.e. type of cancer and type of treatment). Projected universe numbers were validated against secondary sources such as the Surveillance, Epidemiology, and End Results (SEER), Globocan, and Cancer Research UK databases. The Global Oncology Monitor© is validated every 2 years using market sizing studies to ensure that the size and representativeness of the physician sample reflects the wider population of treating physicians.

The analyses were based on the projected estimates for the prevalence of the total number of chemotherapy treatments classified as HEC (including AC) and carboplatin-based, i.e., therapies requiring prophylaxis with NK_1_RA-based regimens according to antiemetic guidelines. Data on prescribed antiemetic regimens for acute CINV prophylaxis are presented.

MASCC/ESMO antiemetic guidelines were used for chemotherapy emetic risk classification, in which the AC combination is classified as HEC, and carboplatin-based regimens are classified at the high end of moderately emetogenic chemotherapy (MEC) [12, 17]. Thus, HEC treatments included cisplatin-based, AC-based, and other HEC therapies. Carboplatin-based therapies were also included if the area under the concentration–time curve (AUC) was 4 mg/mL or greater as established by some guidelines [9, 10, 13, 15], and because these therapies are the same as those recommended in HEC treatments despite their classification as MEC. Guideline adherence was defined according to MASCC/ESMO antiemetic recommendations from 2016 [12], which was the latest updated version at the time the survey was conducted.

### Statistical analysis

Data were analysed using IBM SPSS Data Collection Survey Reporter, version 6.0.1.

## Results

### Study participants and study population

A total of 107 experts were screened. Thirty-four were excluded, because medical records on antiemetic administration were not available. A final sample of 73 physicians was analysed in this study. As shown in Table 1, most were oncologists (49%) from university teaching hospitals (81%) in urban areas (96%). The most frequently treated cancer type was breast cancer (23%) followed by colorectal cancer (20%).

**Table 1:** Baseline characteristics and demographics of survey respondents

Characteristic	Total respondents (*N* = 73)
*Specialty, n (%)*	
Medical oncology	36 (49)
Hematology/oncology	14 (19)
Urology	11 (15)
Dermatologist	4 (5)
Hematology	4 (5)
Neurologist	2 (3)
Other	3 (4)
*Hospital setting, n (%)*	
Urban	70 (96)
Rural	2 (3)
Suburban	1 (1)
*Hospital type, n (%)*	
University/teaching hospital	59 (81)
General	11 (15)
Private hospital	2 (3)
Office/private clinic	1 (1)
*Region—area, n (%)*	
Madrid	23 (31)
Cataluña	13 (18)
Andalusia	10 (13)
Aragon	7 (9)
Galicia	4 (6)
Castile and Leon	4 (6)
Navarre	3 (4)
Castilla la Mancha	2 (3)
Comunidad Valenciana	2 (3)
Extremadura	2 (3)
Cantabria	1 (1)
Principality of Asturias	1 (1)
Region of Murcia	1 (1)
Tumor type, % of chemotherapy treatments*, n (%)	
Breast	17 (23)
Colorectal	15 (20)
Non-small cell lung cancer	6 (8)
Non-Hodgkin’s lymphoma	4 (6)
Urinary and bladder	3 (4)
Ovarian	3 (4)
Pancreas	2 (3)
Other	23 (32)

^*^Administered by physicians to 9529 patients (weighted = 202,084). Percentages are calculated based on the total number of patients

**Fig. 1:**
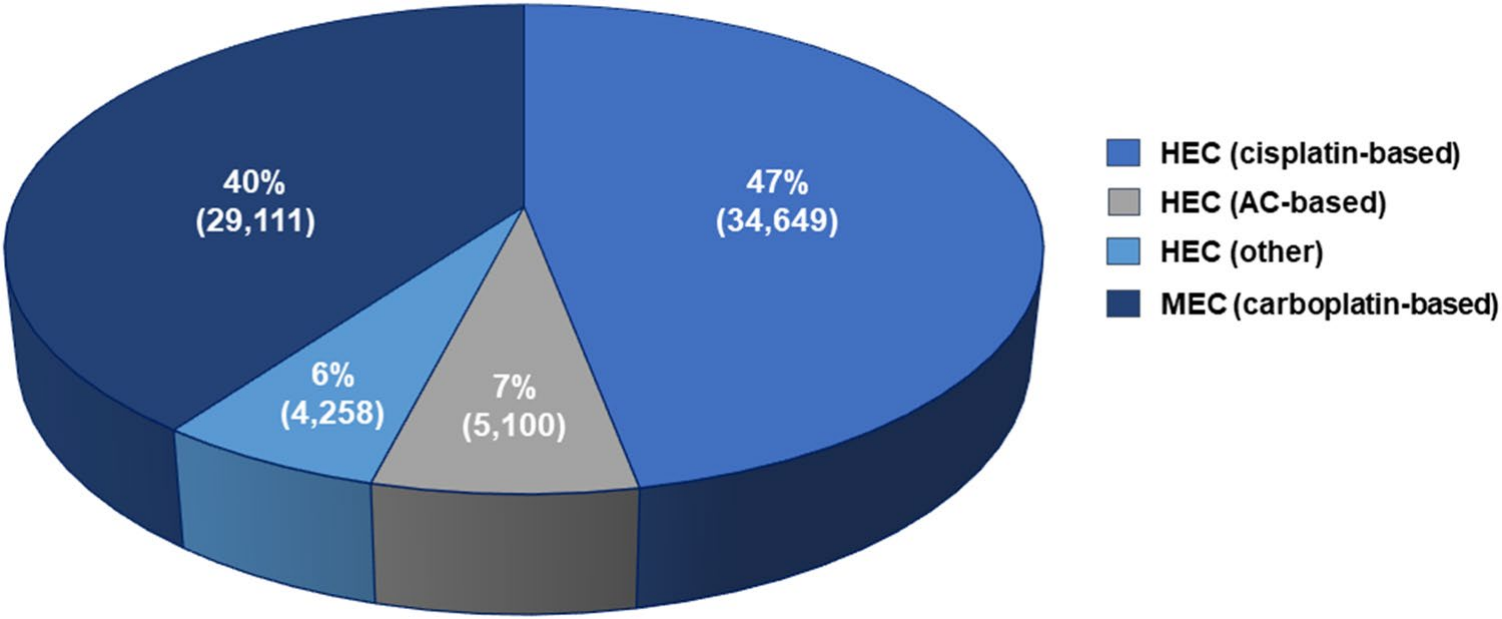
Distribution of chemotherapeutic regimens according to their emetic risk. *HEC* highly emetogenic chemotherapy, *MEC* moderately emetogenic chemotherapy, *LEC* low emetogenic chemotherapy, *Carbo* carboplatin

Physicians reported data from a total of 9519 patients, who were estimated to be representative of 202,084 patients. Of these, a total of 200,014 received chemotherapy treatments associated with any emetic risk (HEC, MEC, LEC and minimal emetogenic chemotherapy). HEC (including MEC carboplatin-based regimens; henceforth HEC + carbo) was administered to 73,118 (36%) patients, the most frequent treatment being cisplatin-based therapy (*n* = 34,649, 47.38%) (**Fig. ****1**).

### Use of NK_1_RA-based antiemetic regimens for patients treated with HEC

**Fig. 2:**
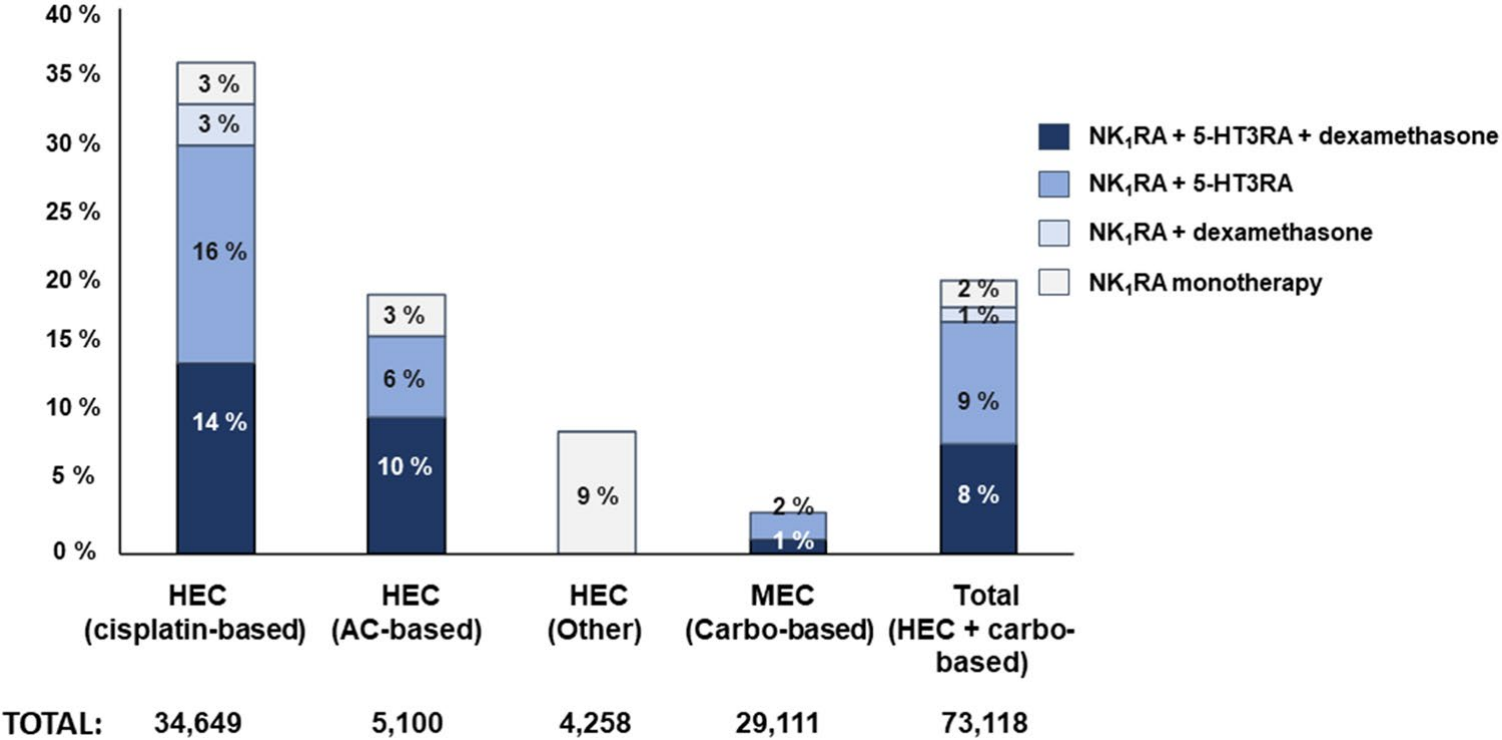
Distribution of NK_1_RA-based regimens according to the different HEC regimens

Among the total number of patients who received HEC + carbo chemotherapy, NK_1_RAs (monotherapy or in combination with 5-HT_3_RAs and/or dexamethasone) were used in the acute phase as prophylaxis for CINV in 14,762 (20%) patients and 5-HT3RAs in monotherapy were used in 51,274 (70.13%) patients. Treatments other than NK_1_RAs or 5-HT_3_RAs were prescribed to 893 (1%) patients. Finally, in 8.46% of all HEC + carbo treatments (6189 patients), no antiemetic prophylaxis was administered for the prevention of CINV. Specifically, the combination of NK_1_RA with 5-HT3RA and DEX as recommended by the international guidelines was reported in 5849 (8%) patients, while 6580 (9%) patients received only NK_1_RA plus 5-HT3RA. The distribution of NK_1_RA-based regimens according to the different HEC + carbo treatments is shown in Fig. 2.

**Fig. 3:**
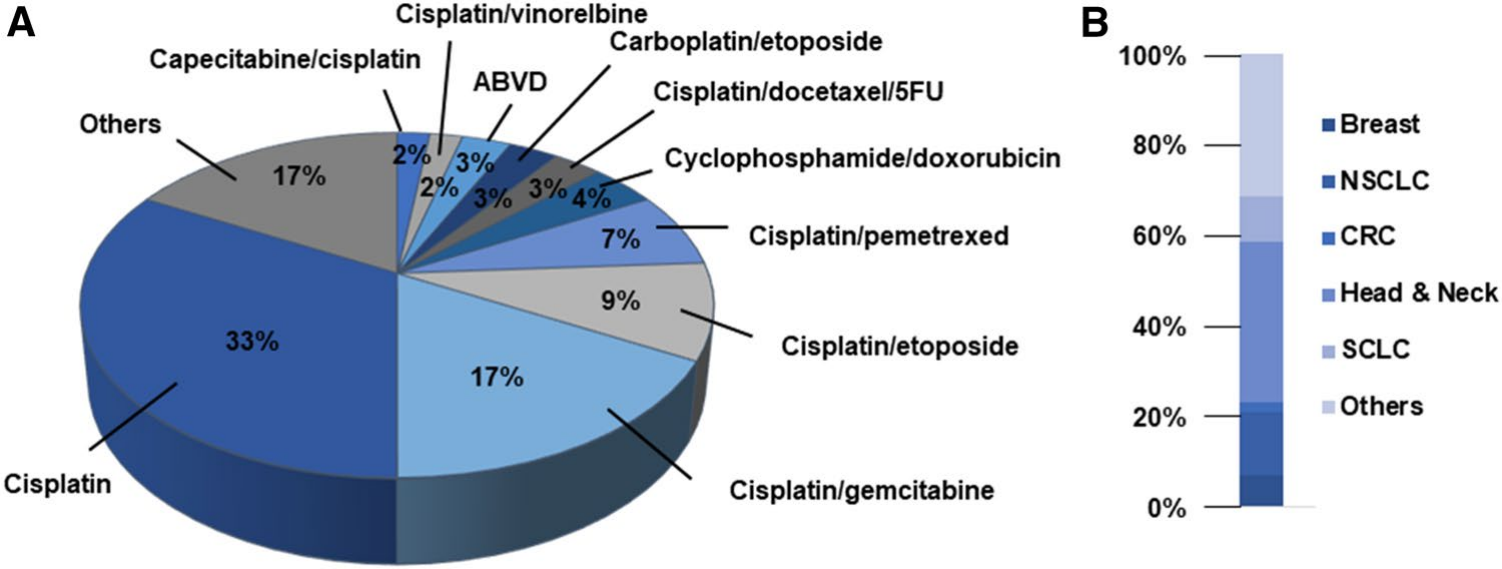
Top ten chemotherapy regimens received by patients treated with NK_1_RA-based prophylaxis (day 1) (**a**) and distribution of regimens according to tumour sites (**b**). *5FU* 5-fluorouracil, *ABVD* doxorubicin, bleomycin, vinblastine and dacarbazine

Among the ten most frequent chemotherapeutic regimens for which NK_1_RA-based prophylaxis was prescribed, cisplatin-based regimens (47.38%) were the most common. Cisplatin alone or in combination with gemcitabine were the treatments most frequently administered to patients receiving NK_1_RAs for the prevention of acute CINV (33% and 17%, respectively) (Fig. 3a). Chemotherapy distribution according to cancer types is shown in Fig. 3b.

### Physicians’ perception of the emetogenic risk of HEC chemotherapy

**Fig. 4:**
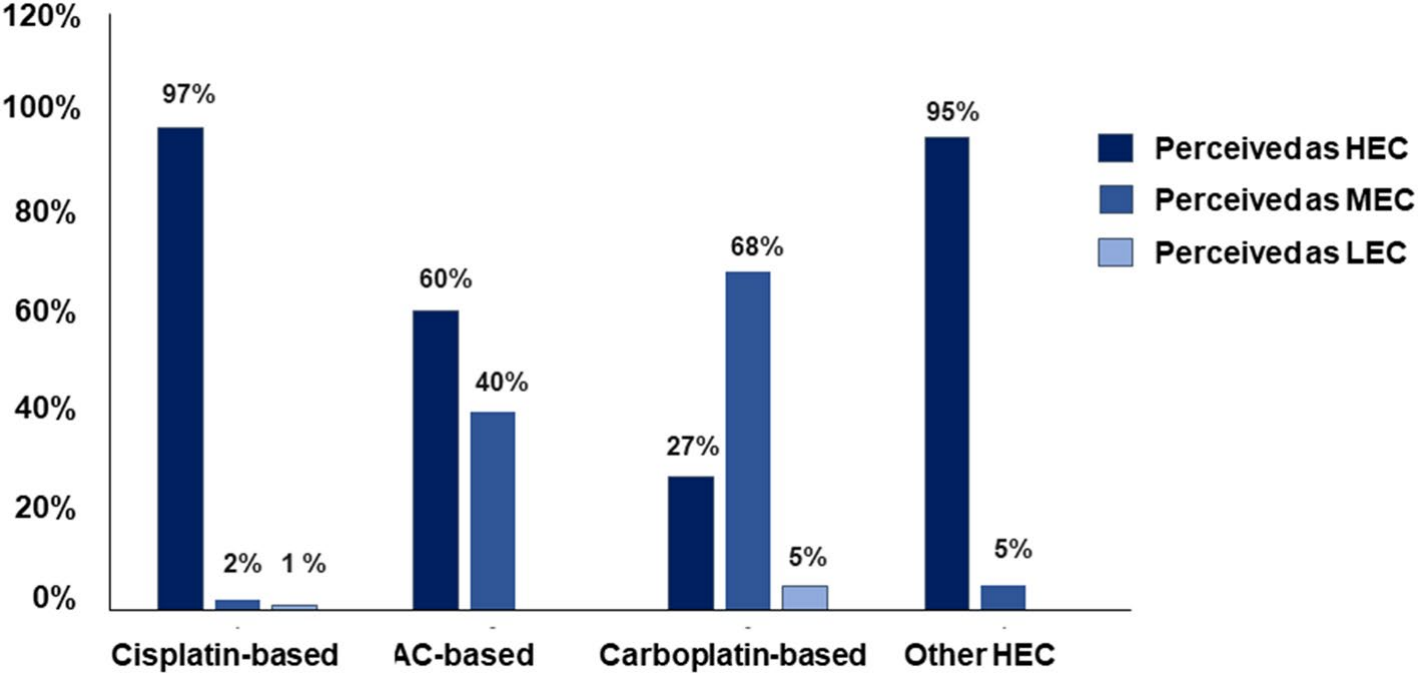
Emetogenic risk of chemotherapy as perceived by physicians who prescribe NK_1_RAs. *HEC* highly emetogenic chemotherapy, *MEC* moderately emetogenic chemotherapy, *LEC* low emetogenic chemotherapy, *AC* anthracycline-cyclophosphamide

Physician’s perceptions vary across the different regimens: study participants defined 97% of cisplatin-, 60% of AC- and 27% of carboplatin-based therapies, as well as 95% of regimens not based on carboplatin or cisplatin as HEC (Fig. 4). Importantly, among physicians who prescribed NK_1_RAs in the HEC + carbo setting, 27,512 (37.63%) perceived these chemotherapy treatments as HEC, 35,290 (48.26%) as MEC, and 5,298 (7.25%) as LEC.

## Discussion

CINV is a common and distressing side effect that, in the absence of antiemetic prophylaxis, occurs in more than 90% of patients receiving HEC and in 30–90% of those receiving MEC [9, 10, 13]. At present, several antiemetic therapies are available and recommended in international antiemetic guidelines. However, up to 61% of patients receiving antiemetic therapy have reported CINV, suggesting poor control that generates a considerable overall public health burden attributable to cancer and its treatment [18–22]. In this study, we have shown important data regarding the use of antiemetics in the real-world setting in Spain.

First, our results show that, despite N_1_KRAs being used mainly as prophylaxis for acute CINV in the HEC setting, the percentage of patients receiving this treatment remained low, with only 29% being prescribed these antiemetics. These results are remarkable, especially if we take into account that since 2004, N_1_KRAs in association with 5-HT3 Ras and corticosteroids have been included in the MASCC/ESMO guidelines for the management of CINV in patients receiving HEC and AC (considered MEC at that time) [23]. Importantly, low adherence to the 2004 guidelines and subsequent updates up to 2016 has been consistently reported in other studies conducted in Spain [24, 25], confirming a trend that appears to be continuing. Adherence to guidelines has been reported not only in Spanish studies, but also in other observational studies conducted in Europe and USA [26–28], including surveys carried out among oncologists [29] and oncology nurses [30, 31].

In the updated version of the MASCC/ESMO guidelines published in 2017 [12], NK_1_RA–5-HT_3_RA–dexamethasone triplet prophylaxis was established as the standard treatment for patients receiving HEC + carbo, including carboplatin-based regimens. Our study shows limited use of the triplet in the Spanish population: only 8% of the patients receiving HEC were treated with this combination. Prescriptions were mainly for cisplatin-based regimens (14%), while the lowest rate for use was in carboplatin-based therapies (1%). The low percentage in the latter case may be explained by the short period between the inclusion of the recommendations in the 2017 guidelines and the time of performance of this study, which may have been insufficient to allow for the total integration and implementation of these practices into clinical routine.

This persistently low adherence to international guidelines may be due to different reasons. Despite the high level of awareness of the recommendations shown by oncologists participating in a large survey conducted in Italy [29], a predominant barrier to their application appeared to be an underestimation of the emetogenic potential of chemotherapy, leading to the utilisation of weaker antiemetic regimens than required. Oncology nurses also identified physicians’ preference as a main cause for poor adherence to antiemetic recommendations in another study published in 2018 [30]. Indeed, in our study, a percentage of HEC-treated patients did not receive any antiemetic treatment. Furthermore, both physicians and nurses appear to underestimate the control of acute CINV in patients receiving HEC regimens [32]. In our study, physicians’ perception of the CINV risk among those who prescribed NK_1_RAs revealed that only 37.63% of them identified cisplatin-based regimens as HEC. Our data are in line with previous observations and support the notion that an inadequate perception of chemotherapy-associated emetogenic risk by physicians is a major cause of the low adherence to guidelines, as suggested by other authors [29, 30].

Nevertheless, it is also important to consider the perspective of patients, who tend to underreport CINV [33–35], because they identify it as a sign of chemotherapy effectiveness [36], because they fear that a dose adjustment will be needed, or because they forget to report it if it happens some time before their next medical appointment [35]. Additionally, mistakes/issues in antiemetic administration by patients have also been suggested as a possible reason for low adherence to international recommendations [29].

This study has some limitations. First, the number of chemotherapy treatments has been extrapolated from patient records from a global database, which may lead to possible errors derived from the methodology applied. Second, in this study, only antiemetic use for acute CINV was analysed, so it was impossible to draw conclusions about the delayed phase, in which even lower rates of guideline adherence have been observed [8, 26, 30, 31]. Patients receiving MEC regimens other than carboplatin, such as oxaliplatin, who may be eligible for NK_1_RA-based prophylaxis but for whom no clear consensus has been reached in guidelines were excluded from the analysis. However, our results are sufficiently robust to be able to demonstrate that antiemetics are being used at levels below the recommendations of the international guidelines.

Our results confirm that strategies for improving adherence in patients receiving HEC and carboplatin are urgently needed. These strategies should focus on ensuring that cancer specialists are aware of the most updated recommendations and understand them. Indeed, educational programs including simple diffusion, an “audit and feedback” strategy, and “educational outreach visits” have been seen to modify physician’s behaviour and improve adherence [37]. Seemingly, a multidisciplinary approach in which clinicians, nurses and pharmacists issue standardised antiemetic prescriptions based on chemotherapy type improved adherence at an institutional level [38]. Moreover, the use of protocolised physician order entry systems implemented in routine practice at medical centres may increase compliance. For the patients, approaches to mitigate CINV underreporting would be also helpful. The use of electronic questionnaires and phone- or web-based tools for reporting symptoms and expressing doubts and/or concerns would stimulate patient–clinician communication and help professionals to identify risks more accurately [39, 40]. In this respect, some initiatives have been launched in Spain, such as “Diario NaVIQ”, a mobile application that allows patients to inform healthcare personnel about the impact of nausea and vomiting on their daily life (https://espacioviforpharma.es/nauseas-y-vomitos-inducidos-por-quimioterapia/diario-naviq/). Finally, the desire of some patients to reduce their pill burden, prompting them to take their medication only when symptoms appear, should be taken into account when aiming to improve treatment adherence. In this respect, NEPA is the only fixed combination of an NK_1_RA and a 5-HT3RA and has the simplest administration schedule [41], offering a highly convenient method of administration for most patients. Simple administration schedules would not only facilitate adherence by physicians, but could also prevent patients from making medication errors, a recurring problem during home administration in the delayed phase.

In conclusion, our results show that in Spain, the use of NK_1_RA-based regimens for CINV prevention in patients treated with HEC (including carboplatin-based regimens) does not meet the recommendations of the MASCC/ESMO antiemetic guidelines, and adherence to these guidelines is poor.
